# A Rare Case of Arterial and Venous Thrombosis in COVID-19

**DOI:** 10.7759/cureus.49206

**Published:** 2023-11-21

**Authors:** Shekhar Bhatta, Elina Shrestha, Asmita Sigdel, Narayan Osti, Prakash Upreti

**Affiliations:** 1 Internal Medicine, BronxCare Health System, Bronx, USA; 2 Internal Medicine, St. Vincent Medical Center, Bridgeport, USA; 3 Pediatrics, Comilla Medical College, Comilla, BGD; 4 Surgery, NYC Health and Hospital Harlem, New York City, USA; 5 Internal Medicine, Rochester Regional Health, New York, USA

**Keywords:** pulmonary embolism, dvt, thrombosis, aorta, covid-19

## Abstract

The coronavirus disease 2019 (COVID-19) pandemic has brought attention to the significant risk of thrombotic complications in infected individuals. We present a rare case of a 64-year-old male with COVID-19 who developed bilateral deep vein thrombosis (DVT), pulmonary embolism (PE), and thrombus in the thoracic and abdominal aorta. The patient exhibited common symptoms of COVID-19 and required intensive care unit admission due to respiratory failure. Subsequent investigations revealed thrombi in the lower extremities, pulmonary arteries, and aorta. Prompt anticoagulation therapy was initiated, and vascular surgery consultation was sought. This case highlights the increased risk of venous and arterial thrombotic events in COVID-19 patients and emphasizes the importance of comprehensive management strategies. The interplay of various factors in COVID-19 contributes to a prothrombotic state, necessitating a multi-modal approach to address thrombotic complications. Further research is needed to optimize treatment protocols and improve outcomes for COVID-19 patients with thrombotic complications.

## Introduction

The coronavirus disease 2019 (COVID-19) pandemic has had a significant global impact, with over 765 million cases reported worldwide and more than 6 million lives lost. In the United States, COVID-19 continues to be a major cause of morbidity and mortality. Common symptoms of COVID-19 include fever, cough, fatigue, loss of taste or smell, sore throat, headache, myalgia, and diarrhea [1]. One of the notable complications observed in COVID-19 patients is the development of a prothrombotic state, with approximately 16% of hospitalized patients presenting with venous or arterial thrombosis [2]. While venous thromboembolism is more prevalent in COVID-19 patients, arterial thrombosis also occurs but at a lower frequency of 14.7% vs. 3.9% [3]. Here, we present a rare case of COVID-19 with bilateral deep-vein thrombosis, pulmonary embolism, and thrombus in the thoracic and abdominal aorta.

## Case presentation

A 64-year-old male with a history of congestive heart failure, methadone use, hypertension, diabetes, chronic obstructive pulmonary disease (COPD), and prior intravenous heroin use presented to the emergency department with a three-day history of subjective fever, shortness of breath, intermittent coughing, myalgia, and fatigue. Notably, the patient had not received the COVID-19 vaccine. On arrival, the patient's vital signs were a temperature of 98.8 degrees F, pulse rate of 115 beats per minute, respiratory rate of 18 breaths per minute, blood pressure of 158/93 mmHg, and oxygen saturation of 88% on room air.

The patient's initial blood gas analysis demonstrated hypoxic hypercapnic respiratory failure and lactic acidosis (pH: 7.20, partial pressure of oxygen (PaO2): 50 mmHg, partial pressure of carbon dioxide (PaCO2): 60 mmHg, bicarb: 28 mEq/L, and lactic acid 4 mmol/L). Chest X-ray revealed mild emphysema. Due to the patient's hypoxia, he was placed on bilevel positive airway pressure (BiPAP) with inspiratory support of 10 cmH2O, expiratory support of 5 cmH2O, and fraction of inspired oxygen (FiO2) of 40% for respiratory support and subsequently admitted to the ICU. However, his partial pressure of carbon dioxide (pCO2) worsened on BIPAP, and he became somnolent, necessitating endotracheal intubation for airway protection and respiratory failure. The patient's COVID-19 test returned positive and was started on remdesivir, dexamethasone, and a prophylactic dose of enoxaparin. After two days, the patient's patient's respiratory condition improved, and he was successfully weaned from mechanical ventilation to a nasal cannula.

**Figure 1:**
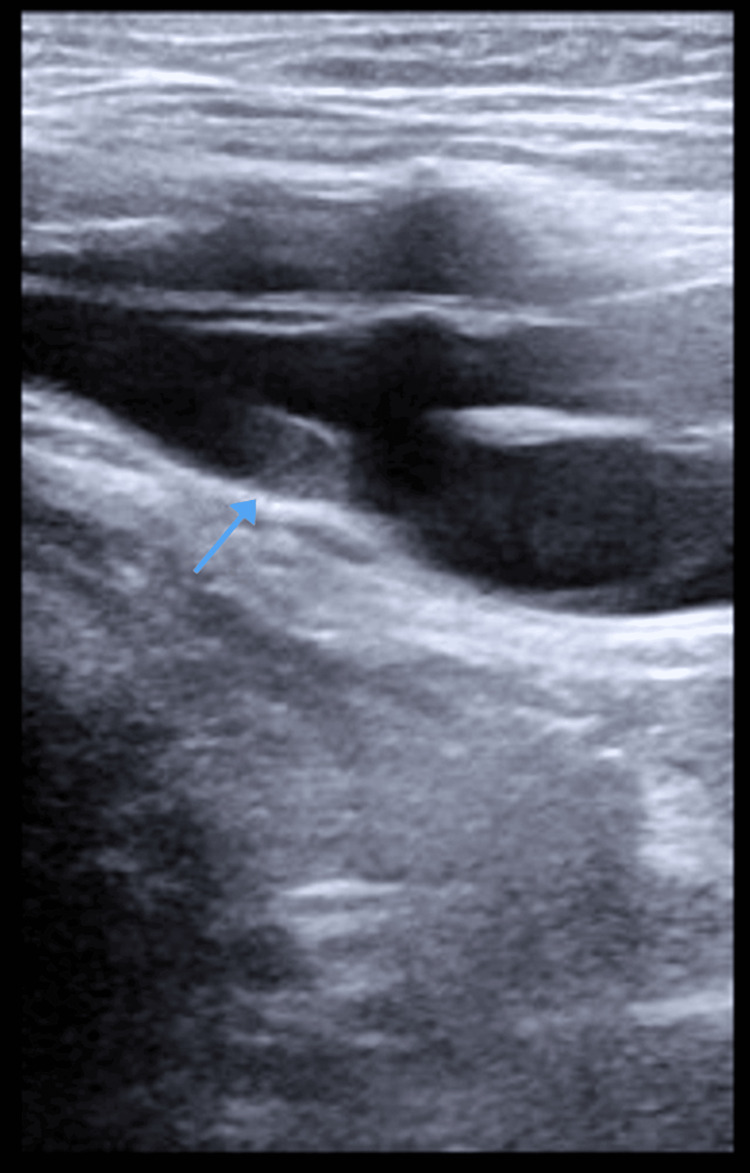
Thrombus in the right common femoral vein (arrow pointing to the thrombus location)

**Figure 2:**
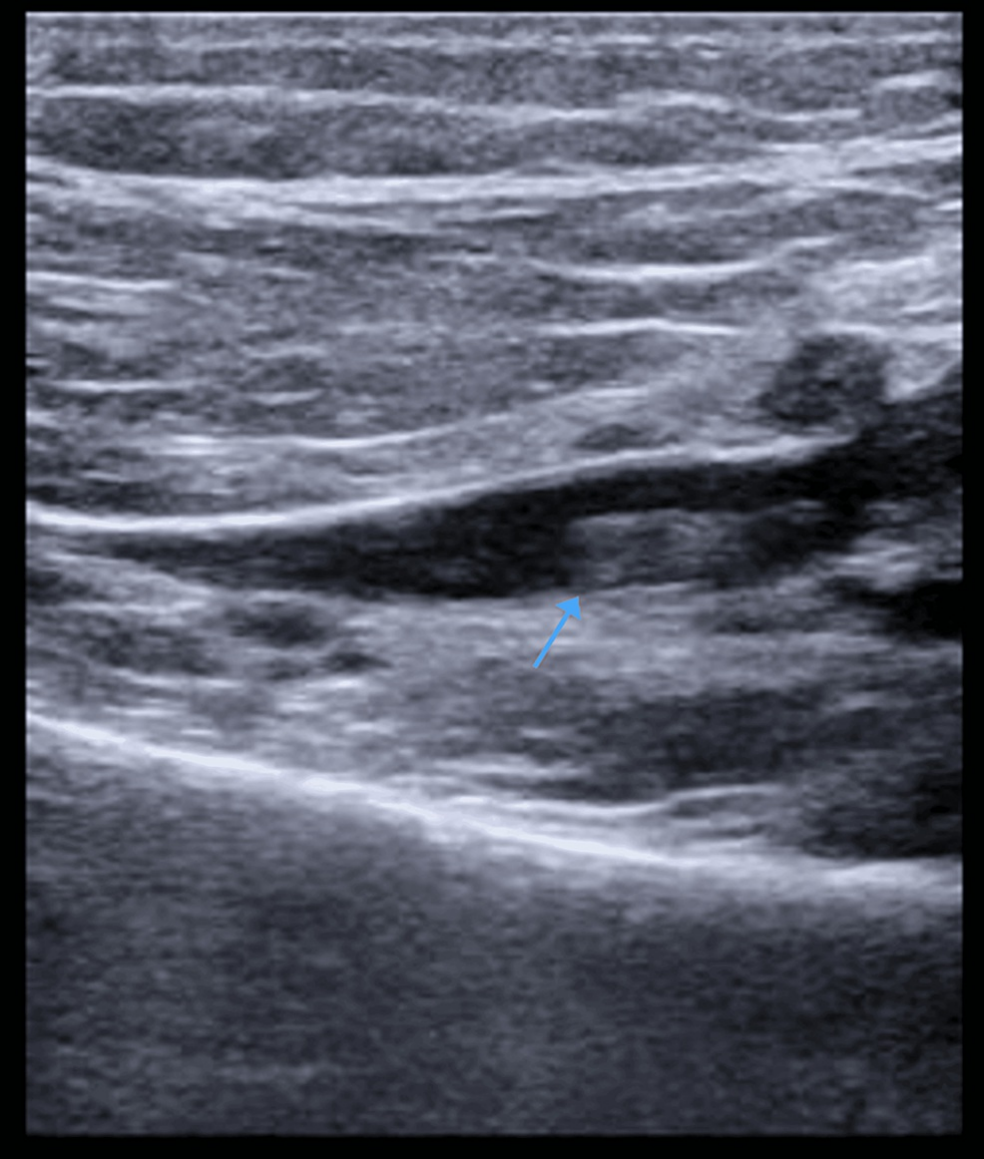
Left popliteal vein thrombosis (arrow pointing to the thrombus location)

Inflammatory markers for COVID-19 were trended every 48 hours and showed increasing D-dimer levels. Due to the patient's worsening D-dimer levels and persistent hypoxia requiring a high-flow nasal cannula with FiO2 up to 50%, further investigations were warranted. An ultrasound of the lower extremities revealed the presence of non-occlusive deep vein thrombi (DVT) within the right common femoral vein and left popliteal vein (Figure 1, Figure 2).

A CT angiogram of the chest was performed, which revealed a pulmonary embolism (PE) with multiple filling defects observed in the left lower lobe segmental pulmonary arteries. Additionally, a prominent intraluminal thrombus measuring 1.51 x 1.11 cm, causing 70% luminal narrowing, was identified in the proximal abdominal aorta. Initially, the patient was started on unfractionated heparin intravenous drip with regular monitoring of the coagulation profile, and the drip was adjusted based on the activated partial thromboplastin time (aPTT).

**Figure 3:**
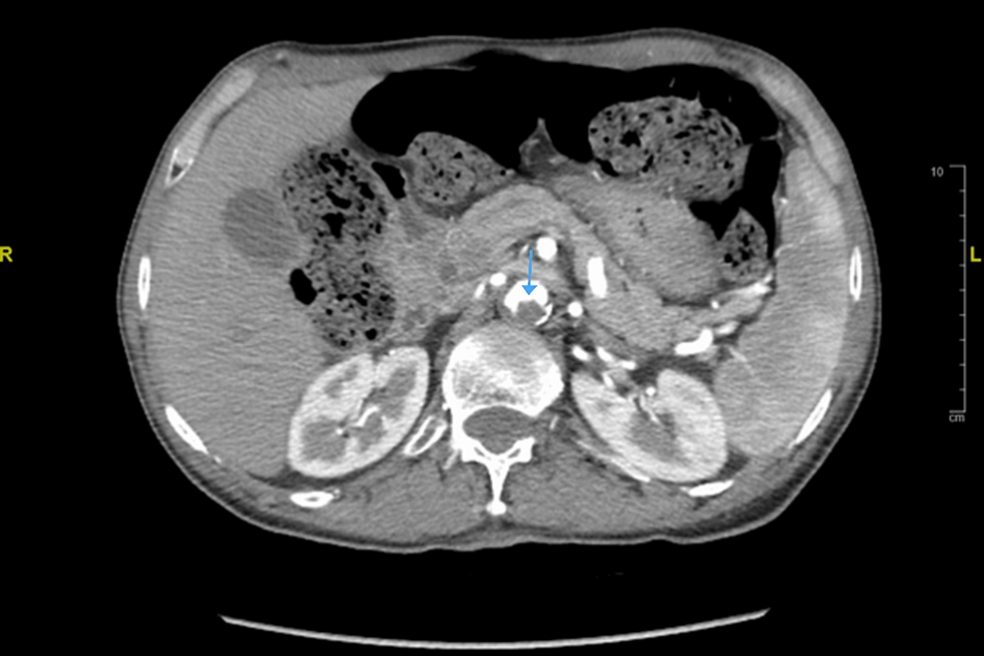
Thrombus in abdominal aorta (arrow pointing to the thrombus location)

Vascular surgery was consulted due to the presence of thromboembolic events. A CT angiogram of the abdomen was performed, revealing an eccentric mural thrombus located in the upper abdominal aorta just below the level of the renal arteries (Figure 3).

The vascular surgery team recommended no acute intervention but advised continuing anticoagulation therapy. The patient was started on a therapeutic dose of apixaban for the treatment. Unfortunately, the patient decided to leave against medical advice on the twelfth day of admission and was lost to follow-up. However, the patient was advised to continue anticoagulation therapy to mitigate the risk of further thromboembolic complications.

## Discussion

Venous thromboembolism is a well-recognized complication of COVID-19, contributing to increased morbidity and mortality. Studies have shown that the prevalence of DVT and PE can be as high as 30% in patients with COVID-19 infection [4]. The prothrombotic state observed in COVID-19 patients results from the complex interplay of various factors triggered by the infection and its sequelae. Proposed mechanisms for increased thrombotic risk in COVID-19 include the release of inflammatory mediators, cytokine storm, endothelial dysfunction, and superimposed infection [5].

Critically ill COVID-19 patients have been found to experience arterial thrombotic events in approximately 4% of cases [6]. Furthermore, a three-fold increase in mortality has been observed in COVID-19 patients who develop arterial thrombotic events during the illness [7]. While arterial thrombotic events are less common than venous thrombosis in COVID-19 patients, they are associated with higher mortality rates. Thrombosis has been reported to involve different arterial systems, including the arteries of extremities, the brain, major arteries of the neck, thorax, and the abdomen [6].

Managing aortic thrombus necessitates a multi-modal approach, with available options including anticoagulation, surgical clot removal, endovascular techniques, and thrombolytic therapy. A meta-analysis comparing anticoagulation and surgical management revealed no significant difference in mortality; however, patients treated with initial anticoagulation had a higher incidence of embolization recurrence [8]. The study analyzed 101 articles with 107 patients; 59 patients were in the open aortic surgery group, whereas 29 patients were in the initial anticoagulation group [8]. Fayad et al. demonstrated that surgical management as a primary therapy yielded better long-term outcomes, with fewer complications compared to anticoagulation alone [9].

## Conclusions

By presenting the rare case of COVID-19 with bilateral DVT, PE, and thrombus in the thoracic and abdominal aorta, we highlight the significance of thrombotic complications in COVID-19 patients and the need for comprehensive management strategies to improve patient outcomes.
